# DNA sensors in metabolic and cardiovascular diseases: Molecular mechanisms and therapeutic prospects

**DOI:** 10.1111/imr.13382

**Published:** 2024-08-19

**Authors:** Hyosang Kwak, Ein Lee, Rajendra Karki

**Affiliations:** ^1^ Department of Biological Sciences, College of Natural Science Seoul National University Seoul South Korea; ^2^ Department of Biomedical Sciences, College of Medicine Seoul National University Seoul South Korea; ^3^ Nexus Institute of Research and Innovation (NIRI) Kathmandu Nepal

**Keywords:** AIM2 and IFI16, cGAS‐STING pathway, DNA‐PK and DDX41, fatty liver and heart diseases, inflammation and cell death, obesity and diabetes

## Abstract

DNA sensors generally initiate innate immune responses through the production of type I interferons. While extensively studied for host defense against invading pathogens, emerging evidence highlights the involvement of DNA sensors in metabolic and cardiovascular diseases. Elevated levels of modified, damaged, or ectopically localized self‐DNA and non‐self‐DNA have been observed in patients and animal models with obesity, diabetes, fatty liver disease, and cardiovascular disease. The accumulation of cytosolic DNA aberrantly activates DNA signaling pathways, driving the pathological progression of these disorders. This review highlights the roles of specific DNA sensors, such as cyclic AMP‐GMP synthase and stimulator of interferon genes (cGAS‐STING), absent in melanoma 2 (AIM2), toll‐like receptor 9 (TLR9), interferon gamma‐inducible protein 16 (IFI16), DNA‐dependent protein kinase (DNA‐PK), and DEAD‐box helicase 41 (DDX41) in various metabolic disorders. We explore how DNA signaling pathways in both immune and non‐immune cells contribute to the development of these diseases. Furthermore, we discuss the intricate interplay between metabolic stress and immune responses, offering insights into potential therapeutic targets for managing metabolic and cardiovascular disorders. Understanding the mechanisms of DNA sensor signaling in these contexts provides a foundation for developing novel interventions aimed at mitigating the impact of these pervasive health issues.

## 1 INTRODUCTION

Pattern recognition receptors (PRRs) are crucial components of the innate immune system that detect pathogen‐associated molecular patterns (PAMPs) and damage‐associated molecular patterns (DAMPs). PRRs are classified based on their cellular location, structural similarities, and ligand specificities. Toll‐like receptors (TLRs) and C‐type lectin receptors (CLRs) are membrane‐bound PRRs, whereas NOD‐like receptors (NLRs), AIM2‐like receptors (ALRs), and RIG‐I‐like receptors (RLRs) are cytosolic.^1^ Certain members of the TLR and ALR families sense both endogenous and microbial DNA. TLRs, which include at least 13 members in mammals (1–10 in humans, 1–9, and 11–13 in mice), are the most extensively studied PRRs.^2^ Almost all TLRs signal through the adaptor MyD88, while TLR3 exclusively uses the TRIF pathway. TLR4 is unique in its ability to signal through both MyD88 and TRIF adaptors. Based on their subcellular localization, TLRs are classified into plasma membrane TLRs and TLRs on intracellular vesicle membranes. TLR1/TLR2, TLR6/TLR2, TLR4, and TLR5 are found on cell surfaces and recognize triacyl lipopeptides, diacyl lipopeptides, lipopolysaccharide (LPS), and flagellin, respectively. TLR9 is located on endosomal membrane and sense cytosine‐phosphate‐guanine DNA (CpG‐DNA).^3^

The ALR family includes four in humans (AIM2, IFI16, PYHIN1/IFIX, and MNDA), and 13 in mice. These proteins possess one or two C‐terminal hematopoietic interferon (IFN)‐inducible nuclear domains with 200 amino acids (HIN‐200) for direct binding to intracellular DNA and an N‐terminal pyrin domain for signaling adaptor recruitment. AIM2, IFI16, and PYHIN1/IFIX are cytoplasmic dsDNA sensors. Additionally, proteins from DExD/H‐box helicase family, such as DHX36 and DDX41, function as DNA sensors. DHX36 binds to G‐quadruplexes^4^ while the central DEAD‐box domain of DDX41 detects bacterial dsDNA and cyclic dinucleotides.^5^, ^6^ DNA damage‐related proteins in the nucleus, such as DNA‐dependent protein kinase (DNA‐PK), meiotic recombination 11 homolog A (MRE11), and Rad50, can also act as cytosolic DNA sensors. DNA‐PK, a heterotrimeric protein complex, consists of Ku70, Ku80 (also known as Ku86), and the catalytic subunit DNA‐PKcs.

DNA sensors have evolved to provide host defense by detecting microbial nucleic acids during infection and activating common downstream signaling pathways that mainly produce type I IFNs.^7^ These sensors are highly expressed in immune cells such as macrophages, neutrophils, monocytes, and dendritic cells, as well as in non‐immune cells like adipocytes, hepatocytes, keratinocytes, and endothelial cells, which regulate tissue homeostasis. However, aberrant activation or expression of molecules in DNA sensing pathways can cause significant tissue damage, leading to auto‐immune diseases, auto‐inflammatory conditions, and cancer.^8^ Chronic low‐grade inflammation, also called metabolic inflammation, is characterized by persistent levels of circulating pro‐inflammatory cytokines, during various metabolic diseases, including obesity, diabetes, fatty liver disease, and cardiovascular disease.^9^ The global prevalence of these metabolic diseases is rising partly due to unhealthy lifestyle habits like overnutrition, alcohol consumption, and sedentary behavior. Metabolic syndrome affects one‐fourth to one‐third of the world population, and poses a significant risk for people developing major diseases such as diabetes, coronary heart disease, and stroke. In the past two decades, research in immunometabolism and inflammaging have gained attention signifying the interplay between inflammation, metabolism, and aging, all of which are directly linked to metabolic disorders. Immune cells within adipose tissue or the liver regulate both tissue homeostasis and metabolic functions, such as lipolysis or insulin signaling.^10^ Conversely, nutrient intake such as sugars, fat, or proteins, and their metabolism modulate immune responses and inflammation. Therefore, metabolic inflammation is orchestrated by cellular and molecular components of the immune response, affecting adipose tissue, liver, pancreas, as well as various organs, contributing to metabolic diseases.^11^

In this review, we summarize the current knowledge on the roles of TLR9, cGAS‐STING, AIM2, IFI16, DNA‐PK, and DDX41 in obesity, diabetes, fatty liver disease, and cardiovascular disease. We discuss the protective or detrimental effects of DNA sensing by immune and non‐immune cells in metabolic function.

## 2 SOURCES OF DNA IN METABOLIC DISORDERS

**FIGURE 1:**
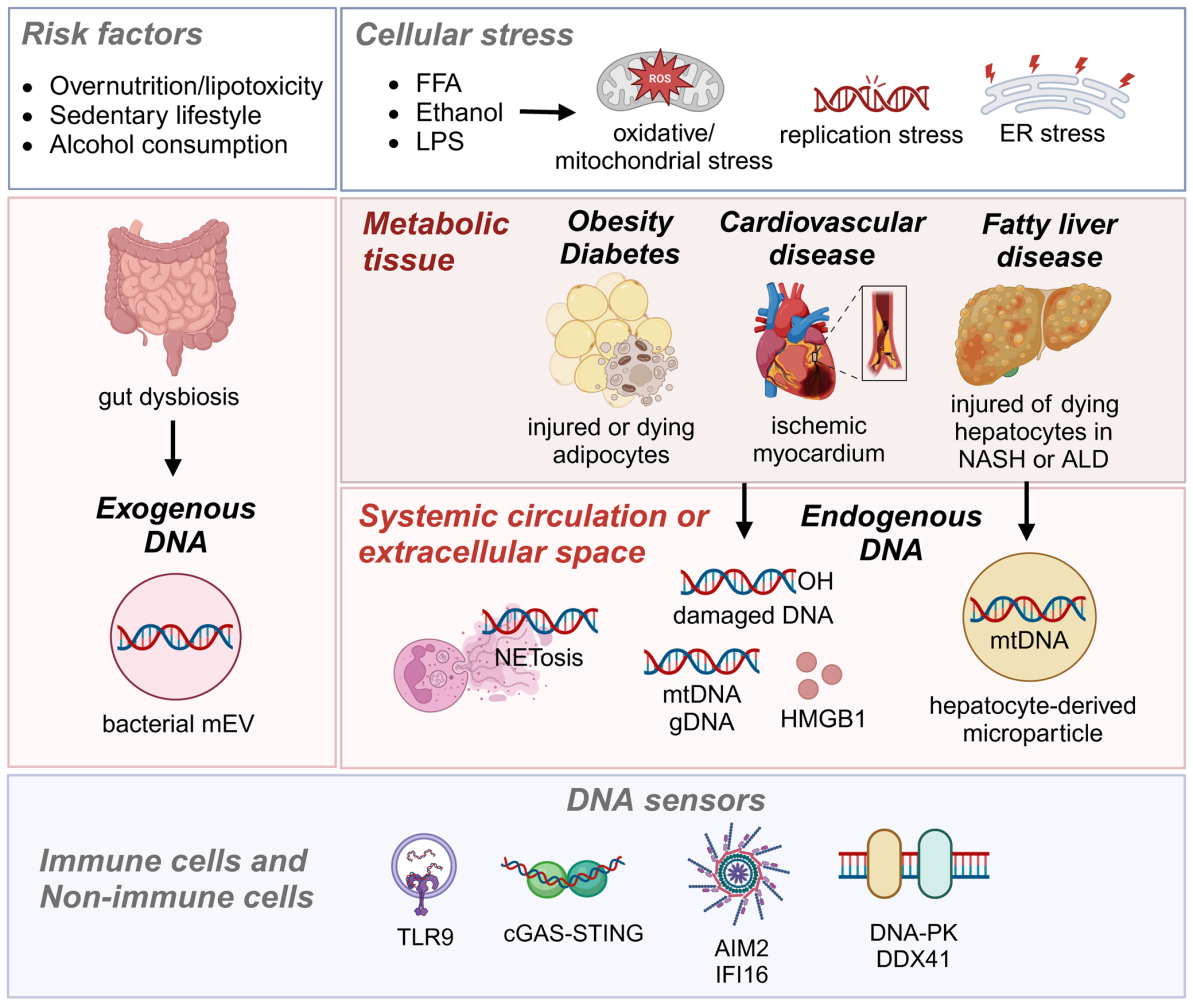
Causes of DNA release and sources of DNA in metabolic tissues. Overnutrition, lipotoxicity, sedentary lifestyle, and excessive alcohol consumption are risk factors for developing metabolic and cardiovascular diseases. These factors trigger oxidative stress, mitochondrial damage, replication stress, and endoplasmic reticulum (ER) stress in various metabolic tissues. Injured or dying adipocytes, cardiomyocytes, and hepatocytes release nuclear DNA (gDNA) and mitochondrial DNA (mtDNA), which activate DNA sensing pathways in an autocrine or paracrine manner. These endogenous DNA fragments, along with other damage‐associated molecular patterns (DAMPs) such as HMGB1, are released into the systemic circulation or extracellular space. Neutrophils release their DNA extracellularly in the form of neutrophil extracellular traps (NETs) during a process called NETosis. Gut‐derived bacterial DNA and endotoxins can enter the systemic circulation and reach metabolic tissues. These endogenous and exogenous DAMPs, including DNA, HMGB1, and endotoxins, activate DNA sensors such as TLR9, cGAS‐STING, AIM2, IFI16, DNA‐PK, and DDX41 in both immune and non‐immune cells.

DNA sensing pathways govern key cellular processes such as energy homeostasis,^12^ apoptosis,^13^ and autophagy.^14^ Activation of these pathways leads to the recruitment of immune cells to metabolic tissues, facilitating phagocytosis to clear cellular debris and metabolites, thereby restoring metabolic function.^15^ However, aberrant DNA sensing can cause persistent inflammation, detrimental to metabolic homeostasis. Both endogenous and exogenous sources of DNA are implicated in metabolic disorders (Figure 1). Exposure to metabolic stressors like saturated free fatty acids (FFAs), lipoproteins, modified cholesterol, glucose, bacterial lipopolysaccharides (LPS), and alcohol triggers endoplasmic reticulum (ER), mitochondrial, replication, and oxidative stress in metabolically active tissues.^16^, ^17^, ^18^, ^19^, ^20^, ^21^, ^22^, ^23^ This stress damages adipocytes, hepatocytes, and cardiomyocytes, leading to the release of endogenous nuclear and mitochondrial DNA into the cytosol, extracellular space, or systemic circulation (Figure 1). During obesity, the downregulation of cytosolic DNase in the liver and adipose tissue contributes to DNA accumulation and activation of DNA sensing pathways.^24^, ^25^ Increased intestinal permeability during obesity elevates serum endotoxin levels and gut‐derived bacterial DNA in the systemic circulation. This gut‐derived exogenous nucleic acid reaches the liver and pancreas via the gut‐pancreas‐liver axis^26^, ^27^ (Figure 1). DNA from various sources is engulfed by tissue‐resident macrophages, where DNA sensors can be activated, leading to production of proinflammatory cytokines and chemokines. This process increases macrophage infiltration into metabolic tissues, perpetuating inflammation and disrupting metabolic homeostasis.^28^, ^29^, ^30^

## 3 TOLL‐LIKE RECEPTOR 9 (TLR9) SIGNALING

TLR9 is a membrane‐bound transmembrane glycoprotein consisting of a transmembrane helix, an extracellular N‐terminal ligand recognition domain, and an intracellular C‐terminal cytoplasmic signal domain. It primarily recognizes pathogen‐derived non‐methylated CpG motifs.^3^ DNA from dying cells can also serve as a physiological ligand for TLR9. Additionally, TLR9 can be activated by endogenous non‐DNA ligands, including heat shock proteins,^31^ antimicrobial peptides,^32^ and high‐mobility group protein B1 (HMGB1).^33^

TLR9 is predominantly expressed in plasmacytoid dendritic cells (pDCs) and B cells but is also found in other immune cells, including T cells, macrophages, and natural killer (NK) cells.^34^ TLR9 transduces signals via MyD88 to activate NF‐κΒ and MAPK signaling pathways, leading to upregulation of several pro‐inflammatory genes. The TLR9 signaling cascade via IRAK1‐TRAF3 results in the production of type I IFNs.^35^ The subsequent choice of signaling pathways, either NF‐κB, MAPK, or IRAK1‐TRAF3, depends on both cell type and the nucleotide composition of the DNA.^36^, ^37^ TLR9‐mediated signaling is implicated in infectious and inflammatory diseases and cancer. Polymorphisms in TLR9 are associated with an increased susceptibility to *Helicobacter* infection.^38^

### 3.1 TLR9 in obesity and diabetes

**FIGURE 2:**
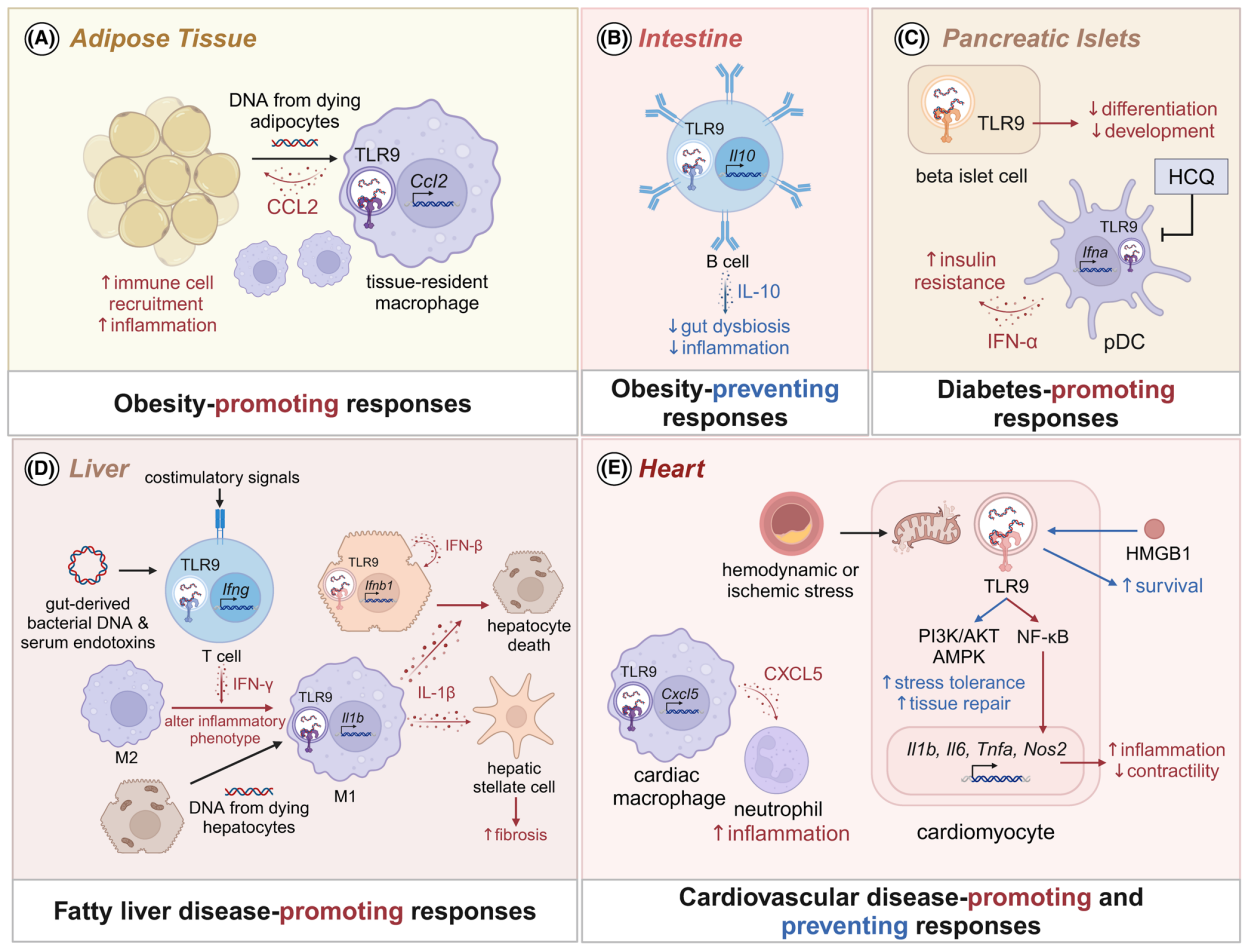
Role of TLR9 signaling in metabolic and cardiovascular diseases. (A) Cell‐free DNA released from dying or damaged adipocytes activates TLR9 in adipose tissue resident myeloid cells. This activation leads to the production of chemokine CCL2, which in turn promotes the recruitment of additional inflammatory cells, thereby amplifying inflammatory responses within the adipose tissue. (B) In humans, TLR9 is primarily expressed on B cells and plasmacytoid dendritic cells (pDCs). TLR9 activation in regulatory B cells within peripheral lymphoid tissues triggers the production of the anti‐inflammatory cytokine IL‐10, which suppresses gut dysbiosis and contributes to the reduction of obesity. (C) TLR9 inhibits beta islet cell differentiation and development. The antimalarial drug hydroxychloroquine (HCQ) exhibits anti‐diabetic effects by inhibiting TLR9‐mediated type I IFNs production in pDCs. (D) In the liver, gut‐derived bacterial DNA and serum endotoxins activate TLR9 in T cells, leading to the production of IFN‐γ, which polarizes liver‐resident macrophages (Kupffer cells) toward an inflammatory phenotype. Additionally, DNA from dying hepatocytes activates TLR9 in these macrophages, resulting in the production of inflammatory cytokines such as IL‐1β, which promotes hepatocyte death (via apoptosis and necrosis) and activates hepatic stellate cells (HSCs), exacerbating liver fibrosis. Furthermore, TLR9 signaling in hepatocytes, triggered by mitochondrial (mtDNA), upregulates IFN‐β production and leads to RIPK1‐depdendent hepatocyte death. (E) In the heart, TLR9 signaling in tissue‐resident macrophages produces the chemokine CXCL5, which recruits neutrophils to ischemic myocardial tissue. Hemodynamic or ischemic stress causes mtDNA leakage, activating TLR9 signaling in cardiomyocytes. This activation stimulates NF‐κB, leading to the induction of pro‐inflammatory mediators such as IL‐1β, IL‐6, TNF‐α, and nitric oxide, contributing to cardiac dysfunction. However, TLR9 also activates the PI3K/AKT and AMPK pathways, altering energy metabolism to increase stress tolerance in cardiomyocytes. Additionally, endogenous damage‐associated molecular patterns like high mobility group box 1 (HMGB1) activate TLR9 signaling in cardiomyocytes, promoting myocardial cell survival, cardiac healing, and angiogenesis.

TLR9 is associated with the development and progression of both obesity and diabetes, which are closely interlinked metabolic disorders (Figure 2A–E). Single nucleotide polymorphisms (SNPs) in TLR9 are associated with susceptibility to obesity and diabetes.^39^ These genetic variations can influence TLR9 expression and function, thereby modulating inflammatory responses and metabolic outcomes. Preclinical studies have shown both beneficial and detrimental roles of TLR9 signaling in the development of obesity and diabetes. In a high‐fat diet (HFD)‐induced mice model of obesity, *Tlr9* ^−/−^ mice gain more body weight and exhibit glucose intolerance and insulin resistance compared to wild type (WT) mice, indicating a beneficial role of TLR9 signaling in obesity development.^40^, ^41^ Compared to WT mice, adipose tissue of *Tlr9* ^−/−^ mice has an increased number of M1 macrophages and TH1 cells, which contributes to elevated levels of pro‐inflammatory cytokines and chemokines.^41^ This suggests that TLR9 signaling helps restrain adipose tissue inflammation and mitigate obesity development. Deletion of TLR9 in B cells reduces interleukin 10 (IL‐10)‐producing B cells and increases IFN‐γ producing T cells^40^ (Figure 2B). B cells without TLR9, or B cells treated with a TLR9 antagonist, show decreased expression of interferon‐regulatory factor 4 (IRF4) and IL‐10, suggesting that TLR9 is necessary for the upregulation of IRF4 and IL‐10 in B cells, contributing to anti‐inflammatory responses. Immunodeficient mice in both specific pathogen‐free or germ‐free conditions receiving splenocytes or fecal microbiota from mice lacking TLR9 in B cells gain more body weight and develop insulin intolerance compared to those receiving the same from TLR9‐sufficient mice, indicating that TLR9 in B cells shapes gut microbiota and modulates inflammatory responses.^40^ This immunomodulatory function of TLR9 is attributed to B cell‐production of IL‐10,^40^ a cytokine which is particularly important in suppressing inflammation and maintaining gut homeostasis^42^ (Figure 2B).

Some studies have reported detrimental effects of TLR9 signaling in obesity. Plasma from HFD‐fed mice contains increased levels of cell‐free DNA,^28^, ^43^ possibly because of damaged adipocytes or increased NETosis.^44^ DNA from dying adipocytes can trigger TLR9 activation in pDCs and adipose tissue resident macrophages to induce CCL2 expression,^28^ which possibly further fuels the inflammatory milieu in the adipose tissue by recruiting inflammatory cells (Figure 2A). Indeed, adipose tissue of HFD‐fed mice lacking CCL2 binding receptor (*Ccr2* ^−/−^ mice) exhibit reduced macrophage infiltration and inflammation, improving insulin sensitivity, glucose tolerance, and suppressing obesity.^45^ Notably, the phenotypes in these studies seem to depend on TLR9 expression in macrophages,^43^ whereas in humans, TLR9 expression is largely restricted in pDCs and B cells.^46^ Administration of hydroxycholorquine (HCQ), an antimalarial drug with inhibitory effects on endosomal TLRs including TLR9,^47^, ^48^ improves insulin sensitivity,^49^, ^50^ potentially offering a therapeutic avenue for obese individuals. The anti‐diabetic effect of HCQ could be through suppression of TLR9‐mediated type I IFN, which contributes to insulin resistance^51^ (Figure 2C).

TLR9 has contrasting roles in type 1 diabetes (T1D). Despite the reduced number of pDCs in T1D patients, increased TLR9‐mediated IFN‐α production is shown to predict the potential risk of T1D development in first‐degree relatives.^52^ TLR9 in B cells, however, stimulates the expansion of IL‐10 secreting B cells, which promotes T1D development in non‐obese diabetic (NOD) mice^53^ (Figure 2B). In the pancreas, TLR9 negatively regulates beta cell growth and contributes to the development of T1D^54^ (Figure 2C). Therefore, the role of TLR9 in pDCs, B cells, NK cells, and pancreatic beta cells is largely dependent on the cell type, leading to either beneficial or detrimental outcomes for T1D.

### 3.2 TLR9 in fatty liver disease

TLR9 expression in the liver is positively correlated with the severity of steatosis in patients with non‐alcoholic fatty liver disease (NAFLD), suggesting its role in pathogenesis.^55^ Patients with nonalcoholic steatohepatitis (NASH) exhibit high levels of mtDNA in hepatocyte‐derived microparticles,^56^ which were shown to activate macrophages via TLR9 in a mouse model of NASH.^43^, ^57^ *Tlr9* ^−/−^ mice fed with a choline‐deficient L‐amino‐defined diet for induction of NAFLD show reduced steatohepatitis and liver fibrosis compared to WT mice, indicating that TLR9 is a driver for fatty liver disease. TLR9 signaling‐dependent IL‐1β secretion by Kupffer cells promotes lipid accumulation, hepatocyte death, and hepatic stellate cell (HSC) activation, driving steatosis and fibrosis^58^ (Figure 2D). TLR9 agonists stimulate liver fibrogenesis by inducing up‐regulation of TGF‐β1 and collagen in HSCs.^24^ As blocking TLR9 signaling in diseased mice could reverse NASH by improving steatohepatitis, fibrosis, and insulin resistance,^43^ it serves as a promising target for therapeutic intervention.

Both apoptotic and necroptotic hepatocyte death, which are potent sources of TLR9‐sensed DNA, contribute to NASH progression. For example, disruption of RIPK3, a key necroptosis signaling molecule, attenuates inflammation, liver injury, and fibrosis in experimental mice models of NASH.^59^, ^60^ TLR9 signaling provides a feed‐forward loop by sensing DNA from dying cells, and releasing cytokines that are able to sensitize cells to undergo cell death (Figure 2D). Accumulation of cytosolic DNA due to reduced DNase II activity triggers aberrant TLR9 signaling, upregulating IFN‐β expression and inducing RIPK1‐dependent necroptotic hepatocyte death, thereby driving liver fibrosis and NAFLD.^56^ In addition to mtDNA from dead hepatocytes, exogenously sourced gut‐derived circulating bacterial DNA detected in patients with fatty liver disease can alternatively trigger TLR9 and drive liver fibrosis^61^, ^62^ (Figure 2D). TLR9 signaling also serves as a co‐stimulatory signal for T cells to produce IFN‐γ,^63^ a potent mediator of cell death and inflammation^64^, ^65^ (Figure 2D).

TLR9 signaling plays a crucial role in the progression of fatty liver disease by promoting inflammation, steatosis, and fibrosis through its interactions with hepatocytes, Kupffer cells, and hepatic stellate cells. Inhibition of TLR9 signaling may offer a promising therapeutic strategy to mitigate these pathological processes.

### 3.3 TLR9 in cardiovascular disease

Inflammatory responses mediated by TLR9 signaling contribute to cardiomyocyte dysfunction under hemodynamic and ischemic stress conditions. In isolated ventricular cardiomyocytes, TLR9 triggers NF‐κΒ activation for upregulation of inducible nitric oxide synthase (iNOS) and pro‐inflammatory cytokines such as IL‐1β, IL‐6, and TNF‐α, leading to impaired cardiomyocyte contractility^66^ (Figure 2E). Mice with cardiac‐specific deletion of lysosomal DNase II exhibit severe myocarditis, dilated cardiomyopathy, and increased mortality upon challenge with pressure overload. Inhibition of TLR9 signaling attenuates inflammatory responses and the development of cardiomyopathy in DNase II‐deficient mice or WT mice subjected to pressure overload.^67^, ^68^, ^69^ Mitochondrial DNA that escapes degradation by autophagy activates TLR9‐mediated inflammatory responses in cardiomyocytes by triggering NF‐κΒ signaling.^67^, ^68^, ^69^ In the context of cardiac transplant‐mediated ischemia reperfusion injury, TLR9 signaling recruits neutrophils into ischemic myocardial tissue. Tissue‐resident CCR2^+^ monocyte‐derived macrophages mediate this recruitment via a TLR9‐MyD88‐CXCL5 axis,^70^ exacerbating tissue damage and contributing to contractile dysfunction (Figure 2E).

Conversely, TLR9 signaling in macrophages protects against cardiac dysfunction and reduces infarct size during myocardial ischemia/reperfusion (I/R) injury by promoting sustained anti‐inflammatory IL‐10 production.^71^ TLR9 detection of DAMPs like HMGB1, released during hypoxia, aids in myocardial cell survival, wound healing, and angiogenesis, facilitating the repair of infarcted myocardium^72^ (Figure 2E). TLR9 signaling has also been reported to enhance stress tolerance and protect the heart by modulating energy metabolism in cardiomyocytes upon pressure overload and myocardial I/R injury.^71^, ^72^, ^73^, ^74^, ^75^, ^76^ This protective role is not associated with TLR9‐mediated typical inflammatory responses but rather involves energy metabolism that increases stress tolerance against hypoxia in cardiomyocytes. TLR9 activation increases the AMP/ATP ratio, subsequently activating adenosine monophosphate‐activated protein kinase (AMPK).^75^ TLR9 reduces the activity of SERCA2, an ATPase involved in calcium transport between the sarcoplasmic reticulum and mitochondria, leading to decreased mitochondrial ATP levels.^76^ The TLR9 agonist CpG‐oligodeoxynucleotides (CpG‐ODN) confers cardioprotection via phosphoinositide 3‐kinase (PI3K)/protein kinase B (AKT) activation. Furthermore, TLR9 phosphorylation by CpG‐ODN promotes its association with the p85 subunit of PI3K, resulting in AKT and GSK‐3β phosphorylation.^74^ The anti‐hypertrophic effect of cathelicidin‐related antimicrobial peptide also involves PI3K/AKT and AMPK pathways regulated by TLR9,^73^ and inhibition of PI3K/AKT abolishes TLR9‐induced cardioprotection.^74^ TLR9 activating two cross‐inhibitory metabolic pathways simultaneously could be due to the nature of synthetic CpG‐ODNs, which vary in structure and nucleic acid composition, stimulating TLR9 in different cell types with a wide range of dose‐dependent responses.^36^

Altogether, TLR9 signaling in cardiovascular disease exhibits a dual nature. While TLR9‐mediated energy modulation in the myocardium offers cellular protection against cardiac injury, TLR9‐driven inflammation can be detrimental, underscoring the complex and context‐dependent role of TLR9 in heart disease.

## 4 CYCLIC GMP‐AMP SYNTHASE (cGAS)‐STIMULATOR OF INTERFERON GENES (STING) SIGNALING

cGAS binds to dsDNA in a sequence‐independent manner, where the length and bending of dsDNA determine the efficiency of signal transduction. Long and bent dsDNA potently activate cGAS, stabilizing the dimer to form a 2:2 cGAS‐dsDNA complex. Mitochondrial transcription factor A (TFAM) and HMGB1 can induce U‐turns in the DNA, enhancing its immunoreactivity and stimulating cGAS activation.^77^ Upon binding to dsDNA, cGAS dimerizes and becomes enzymatically active, synthesizing the secondary messenger 2′3’‐cyclic GMP‐AMP (cGAMP) from ATP and GTP. cGAMP binds to the adaptor molecule STING, a transmembrane protein located in the ER. STING consists of a transmembrane domain, a cytoplasmic ligand‐binding domain essential for dimerization and cGAMP binding, and a C‐terminal tail containing the TANK‐binding kinase 1 (TBK1) phosphorylation site required for downstream signaling.^78^ Upon cGAMP binding, STING undergoes conformational change and dissociates from the ER. STING can then bind directly to and be phosphorylated by TBK1 or IKK. The STING‐TBK1 or STING/IKK signalosome acts as a scaffold to phosphorylate IRF3 and IκBα, leading to the production of type I IFNs and inflammatory cytokines.^79^

Cyclic dinucleotides produced by bacteria serve as ligands for STING, contributing to immune responses against bacterial infections. The cGAS‐STING pathway is upregulated in several autoimmune diseases, like STING‐associated vasculopathy with onset in infancy (SAVI),^80^ Aicardi‐Goutières syndrome (AGS),^81^ systemic lupus erythematosus (SLE), familial cerebellar lupus erythematosus, and polyarthritis.^82^ Furthermore, this pathway is involved in oncogenesis, with its dysregulation capable of either promoting or suppressing tumorigenesis contingent upon the specific cellular and molecular context.^83^

### 4.1 cGAS‐STING in obesity and diabetes

**FIGURE 3:**
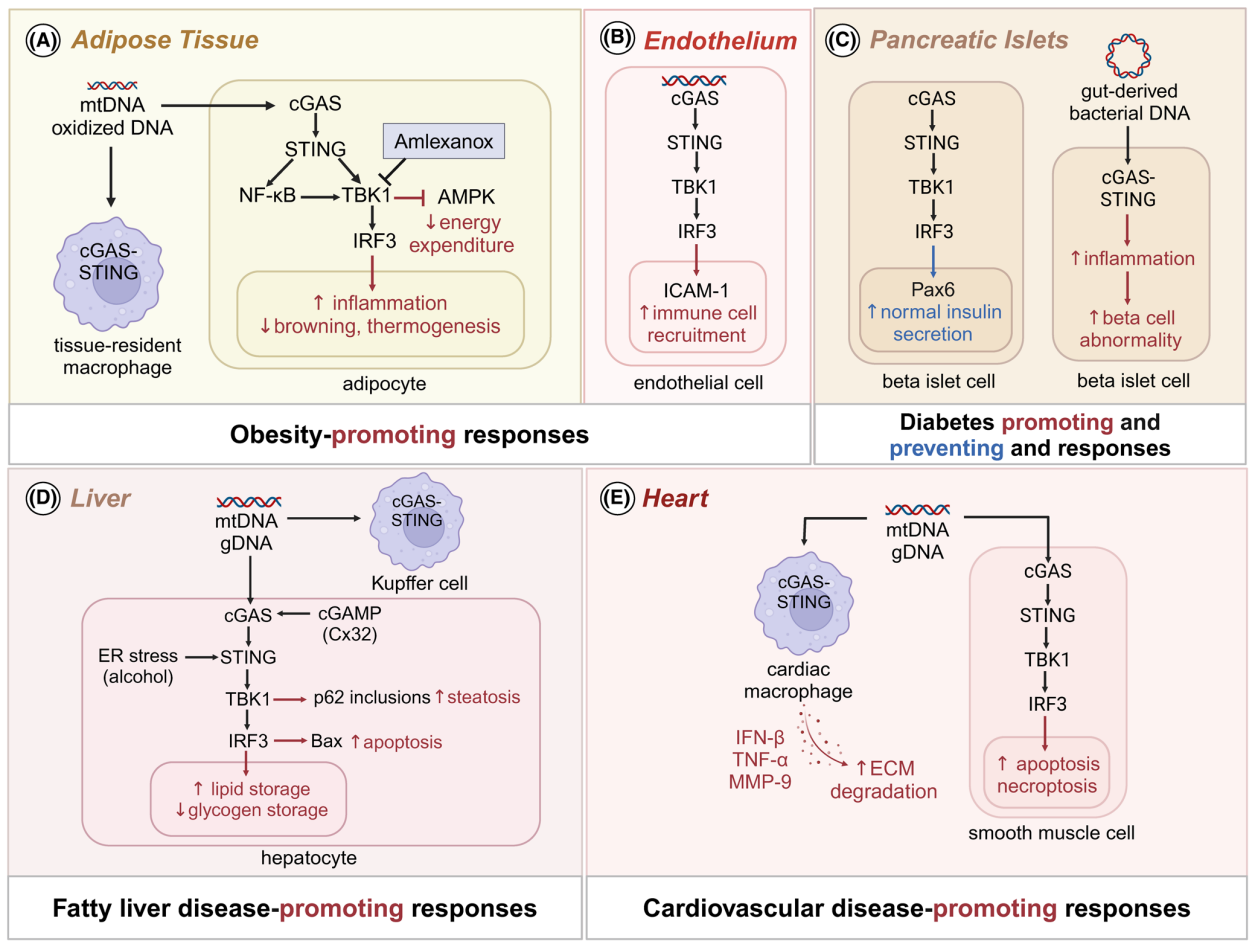
Role of cGAS‐STING signaling in metabolic and cardiovascular diseases. (A) Mitochondrial and oxidized DNA activate cGAS‐STING in adipocytes and macrophages within adipose tissue. The downstream molecule TBK1 inhibits AMPK activation, reducing energy expenditure in adipocytes. IRF3 downregulates genes related to browning and thermogenesis in adipose tissue. The anti‐inflammatory drug amlexanox suppresses obesity and T2D by inhibiting STING‐TBK1 signaling. (B) The transcription factor IRF3 upregulates ICAM‐1 expression in endothelial cells, promoting immune cell infiltration into adipose tissue and exacerbating inflammation. (C) In pancreatic beta cells, cGAS‐STING‐mediated IRF3 activation upregulates the transcription factor Pax6, maintaining normal insulin secretion. However, gut‐derived bacterial DNA can activate cGAS‐STING signaling, leading to the production of inflammatory mediators that cause beta cell dysfunction. (D) Mitochondrial and nuclear DNA activate cGAS‐STING in hepatocytes and Kupffer cells. cGAMP is transferred from injured hepatocytes through Cx32 gap junctions, amplifying apoptotic and inflammatory signals. Alcohol induces ER stress and STING activation. TBK1 phosphorylates p62 into insoluble aggregates, a hallmark of steatotic hepatocytes. IRF3 non‐canonically binds to Bax in the cytosol promoting hepatocyte apoptosis. Additionally, IRF3 alters the expression of hepatic genes related to glucose and lipid metabolism. (E) DNA from injured cardiomyocytes activates cGAS‐STING in cardiac macrophages, leading to the secretion of type I IFNs and TNF. These cytokines induce MMP‐9, resulting in the degradation of the extracellular matrix (ECM) and the loss of smooth muscle cells. Additionally, cGAS‐STING‐mediated IRF3 activation promotes smooth muscle cell death.

cGAS‐STING signaling plays an important role in regulating energy homeostasis and metabolic pathways (Figure 3A–E). Global STING deficiency prevents HFD‐induced adipose tissue inflammation, insulin resistance, and glucose intolerance,^17^ indicating that STING promotes obesity development. Both mitochondrial DNA and oxidized DNA activate cGAS‐STING in adipocytes and macrophages during obesity.^84^ Interestingly, oxidized DNA, which is resistant to DNase‐mediated degradation, is a much more potent activator of the STING‐dependent cytosolic DNA sensing pathway.^85^ In endothelial cells, palmitic acid causes mitochondrial damage and release of DNA, activating TBK1‐IRF3 axis via cGAS‐STING. The transcription factor IRF3 upregulates expression of intercellular adhesion molecule 1 (ICAM‐1) in the endothelium, promoting immune infiltration into the adipose tissue and aggravating adipose tissue inflammation during obesity^17^ (Figure 3B). On the other hand, inflammatory signaling mediated by cGAS‐STING in adipose tissue suppresses thermogenesis, contributing to obesity progression. The effect of cGAS‐STING signaling in thermogenesis has been studied in cold exposed mice. Uncoupling protein 1 (UCP1) is a mitochondrion inner membrane‐resident uncoupler which dissipates energy in the form of heat without producing ATP. Loss of STING promotes UCP1 expression in adipose tissue, and cGAMP stimulation in adipocytes suppresses it. The expression of thermogenic genes including UCP1 is decreased in both inguinal white adipose tissue (iWAT) and brown adipose tissue (BAT) of mice deficient for the fat‐specific disulfide bond A oxidoreductase‐like protein (DsbA‐L), which suffer from aberrant mitochondrial DNA release.^86^ Suppressing cGAS or STING expression in DsbA‐L‐deficient adipocytes reduces TBK1 and IRF3 phosphorylation, concurrently reversing the decrease in UCP1 expression. Furthermore, treatment of DsbA‐L‐deficient adipocytes with amlexanox, a TBK1/IKKε inhibitor, increases UCP1 expression^86^ (Figure 3A).

The kinases downstream of the cGAS‐STING pathway play a major role in the energy homeostasis of metabolic organs. Inhibiting TBK1 with amlexanox improves obesity, insulin sensitivity, and glucose tolerance in HFD‐fed mice, ob/ob mice, and T2D patients.^87^, ^88^ Adipocyte‐specific TBK1 deficiency attenuates HFD‐induced obesity by increasing energy expenditure.^12^ In adipocytes, TBK1 directly inhibits AMPK activity, thereby decreasing lipid oxidation and mitochondrial biogenesis.^12^ However, during calorie restriction, AMPK‐activated ULK phosphorylates TBK1, inhibiting inflammation by degrading NF‐κΒ‐inducing kinase (NIK). TBK1 also associates with mammalian target of rapamycin complex 1 (mTORC1) to suppress its activity, decreasing cellular mitochondrial respiration and increasing systemic fat metabolism and glycolysis.^25^ These findings show the bidirectional role of TBK1 in decreasing energy expenditure during obesity and suppressing inflammation during nutrient deprivation. Interestingly, the alteration of energy homeostasis by TBK1 is independent of IRF3 but dependent on STING.^25^

The role of IRF3, the canonical transcription factor of the cGAS‐STING pathway, remains controversial. IRF3 promotes HFD‐induced adipose tissue inflammation and insulin resistance.^89^ IRF3 is upregulated in the adipocytes of obese mice and humans. IRF3 deletion in obese mice increases the expression of glucose transporter type 4, which improves insulin sensitivity and glucose tolerance. IRF3 downregulates the expression of thermogenic genes in obese mice, suppressing the browning of white fat and decreasing energy expenditure^89^ (Figure 3A). Conversely, other studies show that IRF3 prevents adipose tissue and hepatic inflammation, insulin resistance, and steatosis.^90^, ^91^ In the cytosol, IRF3 was shown to bind to inhibitor of kappa B kinase beta (IKKβ) and represses IKKβ/NF‐κB signaling, uncovering a non‐canonical role of cytosolic IRF3 in preventing NF‐κB‐mediated inflammation during obesity.^90^ Furthermore, *Irf3* ^−/−^ mice develop obesity, insulin resistance, glucose intolerance, and eventually T2D with aging, which is associated with increased inflammatory responses and adipogenesis.^91^

STING distinctly regulates glucose homeostasis in peripheral tissues and β‐cells. In contrast to global STING deficiency that alleviates HFD‐induced insulin resistance and glucose intolerance^17^; β‐cell‐specific STING deletion impairs glucose‐stimulated insulin secretion (GSIS) and causes glucose intolerance.^92^ In the islet cell, STING fine‐tunes the function of the transcription factor pax6 to maintain normal insulin secretion (Figure 3C). STING expression is decreased in the islets of db/db mice and patients with type 2 diabetes (T2D),^92^ suggesting that its loss in the pancreas increases diabetes risk. Bacterial DNA is markedly enriched in the pancreatic β cells of obese hosts, including T2D and obese patients and HFD‐fed WT mice.^26^ Gut‐derived microbial DNA‐containing extracellular vesicles translocate to the pancreas during obesity, causing cGAS‐STING‐mediated chronic islet inflammation and β cell abnormalities^26^ (Figure 3C).

Mitochondrial and oxidized DNA activate cGAS‐STING in adipocytes and macrophages, exacerbating inflammation and reducing thermogenesis, while the downstream kinase TBK1 plays a dual role by inhibiting energy expenditure during obesity and suppressing inflammation during nutrient deprivation. The role of IRF3 is complex, promoting adipose tissue inflammation and insulin resistance in some contexts, while preventing it in others. Overall, the cGAS‐STING signaling pathway intricately intersects with various aspects of energy homeostasis and metabolic regulation, significantly contributing to the pathophysiology of obesity, adipose tissue inflammation, and insulin resistance through multiple mechanisms.

### 4.2 cGAS‐STING in fatty liver disease

The activation of cGAS‐STING in liver cells contributes to the development of both NAFLD and alcoholic liver disease (ALD).^13^, ^21^, ^29^, ^93^ Livers from NAFLD patients display nucleotide pathway deregulation and cGAS/STING gene alteration.^20^ Imbalance in the nucleotide pool in hepatocytes during NAFLD can cause replication stress, leading to activation of the cGAS/STING pathway.^20^ Palmitic acid induces the DNA‐damage response protein p53‐binding protein 1 (53BP1) to localize in the nucleus in hepatocytes,^94^ implying that dietary lipid can cause DNA damage in the liver. The expression of 53BP1 positively correlates with NAFLD severity.^94^ Fatty acid treatment of hepatocytes induces upregulation of STING and IRF3.^93^ Chronic exposure to STING ligands like 5,6‐dimethylxanthenone‐4‐acetic acid exacerbates liver steatosis and inflammation, promoting NASH progression.^29^ Conversely, inflammatory cytokine production from Kupffer cells, triggered by mitochondrial DNA from dying hepatocytes, is attenuated by STING deficiency.^29^ In hepatocytes, saturated fatty acid‐induced lipotoxicity provokes cGAS‐STING‐mediated TBK1 activation which subsequently phosphorylates p62 (Figure 3D). This leads to abnormal accumulation of ubiquitinated protein inclusions that are not able to be degraded, due to interference with autophagic flux by lipotoxic stress.^95^ Hepatocyte‐specific knockout of p62 reverses the formation of such inclusions, and protects against fibrosis during NASH.^96^ Moreover, STING deficiency or TBK1 inhibition by a small molecule suppresses p62 protein inclusions in liver of HFD‐fed mice.^93^ Consistently, TBK1 inhibition reduces fibrotic pathologies.^93^, ^97^

Mice genetically deficient in STING, cGAS, or IRF3 are protected against ALD.^13^ Livers from alcohol‐fed cGAS‐deficient mice show a marked reduction in IRF3 activation and exhibit lower expression of interferon inducible genes (ISGs), similar to levels observed in the liver of *Irf3* ^−/−^ mice, indicating that alcohol‐induced IRF3 activation requires cGAS and STING.^13^ Importantly, mice deficient for cGAS in hepatocytes are protected against ALD, implying pathogenic cGAS signaling in liver parenchyma during ALD. ER stress induced by alcohol causes activation of STING, phosphorylation of IRF3 and hepatocyte death^21^ (Figure 3D). IRF3 promotes hepatocyte apoptosis through a pathway independent of its conventional role as a transcription factor for type I IFNs. IRF3 activation leads to its interaction with pro‐apoptotic B‐cell lymphoma 2 (Bcl2)‐associated X protein (Bax), causing hepatocyte apoptosis,^13^ thereby setting the stage for inflammation and injury during ALD initiation (Figure 3D). Furthermore, gap junctions facilitate intercellular transfer of cGAMP from injured cells to bystander cells, amplifying IRF3 activation across cells.^98^ Blocking intercellular transfer of cGAS‐produced cGAMP through either pharmacologic or genetic ablation of connexin 32 (Cx32) prevents alcohol‐induced IRF3 activation, oxidative stress, inflammation, and liver injury^13^ (Figure 3D).

In conclusion, the cGAS‐STING pathway plays a central role in driving inflammatory and fibrotic responses, as well as hepatocyte apoptosis, leading to the pathogenesis of both NAFLD and ALD. Modulating this pathway offers promising therapeutic potential for the treatment of liver diseases.

### 4.3 cGAS‐STING in cardiovascular disease

The cGAS‐STING pathway is upregulated in SAVI, an autoinflammatory disease of the vascular and pulmonary systems.^80^, ^99^, ^100^ Clinical phenotyping of children with SAVI has revealed three dominant gain‐of‐function mutations in exon 5 of the *STING* (*TMEM173*) gene,^100^ identifying key amino acids in STING‐involved regulation of type I IFN signaling. Hyperactivated STING in peripheral blood mononuclear cells (PBMCs) from SAVI patients causes elevated transcription of *IFNB1* and other STING target genes. When stimulated with cGAMP, fibroblasts and endothelial cells from these patients show increased *IFNB1* transcription, endothelial activation, and apoptosis.^80^

The cGAS‐STING pathway contributes to chronic vascular inflammation and atherogenesis. STING polymorphisms such as SNP R293Q which desensitize innate immune sensing of endogenous DNA protects against cardiovascular risks,^101^ implying STING activation has an adverse effect on cardiovascular health. In apolipoprotein E‐deficient (*Apoe* ^−/−^) mice, cGAMP levels are increased in the aorta. Genetic deletion or pharmacological blockade of STING in these mice reduces atherosclerotic lesions, lipid accumulation, and macrophage infiltration in atherosclerotic plaques.^16^ Atherogenesis in *Apoe* ^−/−^ mice is particularly mediated by STING signaling in macrophages.^16^ Hypercholesterolemia induces DNA damage in macrophages of ApoE‐deleted mice, and this signals to activate STING‐IRF3 in endothelial cells, upregulating ICAM‐1.^17^ Furthermore, IFN‐β stimulation of myeloid cells enhances macrophage‐to‐endothelium adhesion and immune cell recruitment in atherosclerotic lesions by increasing chemotactic factor expression.^102^ STING‐mediated TBK1‐IRF3 and NF‐kB signaling in macrophages also upregulates TNF‐α and IFN‐β expression, fueling inflammatory responses (Figure 3E).

During myocardial infarction (MI), the cGAS‐STING pathway hinders tissue repair through type I IFNs. *cGas* ^−/−^, *Sting* ^−/−^, *Irf3* ^−/−^, and *Ifnar1* ^−/−^ mice exhibit impaired ISG expression in the infarct region and have improved survival post‐MI.^30^, ^103^ Nucleic acids released from dead cardiomyocytes during ischemic injury activate cGAS‐STING signaling in cardiac macrophages, leading to type I IFN secretion. cGAS‐STING activation drives macrophages toward an inflammatory M1‐like phenotype, while cGAS silencing promotes a reparative M2‐like phenotype, enhancing repair, and hemodynamic performance.^103^

cGAS‐STING signaling also plays a role in sporadic aortic aneurysm and dissection (AAD) by causing progressive aortic smooth muscle cell (SMC) loss and extracellular matrix (ECM) degradation (Figure 3E). Cytosolic DNA is detected in SMCs and macrophages of human AAD tissues. Leakage of nuclear and mitochondrial DNA from SMCs triggers its cell death. In addition, released DNA engulfed by macrophages activates STING and IRF3 axis, inducing matrix metalloproteinase‐9 (MMP‐9) expression (Figure 3E). This signaling pathway functions in both SMCs and macrophages during aortic rupture.^104^ Overall, the cGAS‐STING pathway plays a complex and multifaceted role in cardiovascular diseases, driving both harmful inflammation and essential cellular functions.

## 5 ABSENT IN MELANOMA 2 (AIM2) SIGNALING

AIM2 assembles a multiprotein complex inflammasome in response to cytosolic DNA. AIM2 contains a positively charged C‐terminal HIN‐200 domain that interacts with the negatively charged DNA sugar phosphate backbone and an N‐terminal PYD domain, a member of the death domain‐fold family that interacts with the common inflammasome adaptor ASC. Binding of the dsDNA sugar‐phosphate backbone to the HIN‐200 domain relieves PYD for self‐oligomerization and interaction with ASC and pro‐caspase‐1.^105^, ^106^ Autocleavage of pro‐caspase‐1 generates active caspase‐1, which cleaves pro‐IL‐1β and pro‐IL‐18 into their mature forms and also cleaves gasdermin D (GSDMD). The N‐terminal of GSDMD oligomerizes on the plasma membrane to create a lytic pore that facilitates the release of mature cytokines and cellular DAMPs in a lytic cell death process called pyroptosis.^106^ AIM2 binds DNA independent of its sequence, while efficiency of inflammasome assembly depends on DNA length. dsDNA longer than 70 bp is able to initiate inflammasome assembly with maximal efficiency achieved by those between 250 and 300 bp.^107^ AIM2 also functions in the nucleus, where it is recruited to chromatin sites of radiation‐induced DNA damage.^108^ AIM2‐dependent inflammasome signaling is critical for host defense against DNA viruses and cytosolic bacteria.^105^, ^109^

Interestingly, AIM2 also has inflammasome‐independent roles. AIM2 forms a complex with DNA‐PK to repress AKT and c‐Myc phosphorylation, reducing cell proliferation and promoting cell death in colon tumorigenesis, mediated by PTEN.^110^, ^111^ In addition, AIM2 regulation of AKT3 activity via DNA‐PK inhibits inflammatory antiviral signal transmission in microglial cells.^112^ AIM2 also inhibits stem cell proliferation by stabilizing Wnt, thus preventing gut dysbiosis and colorectal tumorigenesis.^113^ In regulatory T cells, AIM2 associates with RACK and PP2A to prevent AKT and mTORC1 activation, restraining autoimmune diseases.^114^

### 5.1 AIM2 in obesity and diabetes

**FIGURE 4:**
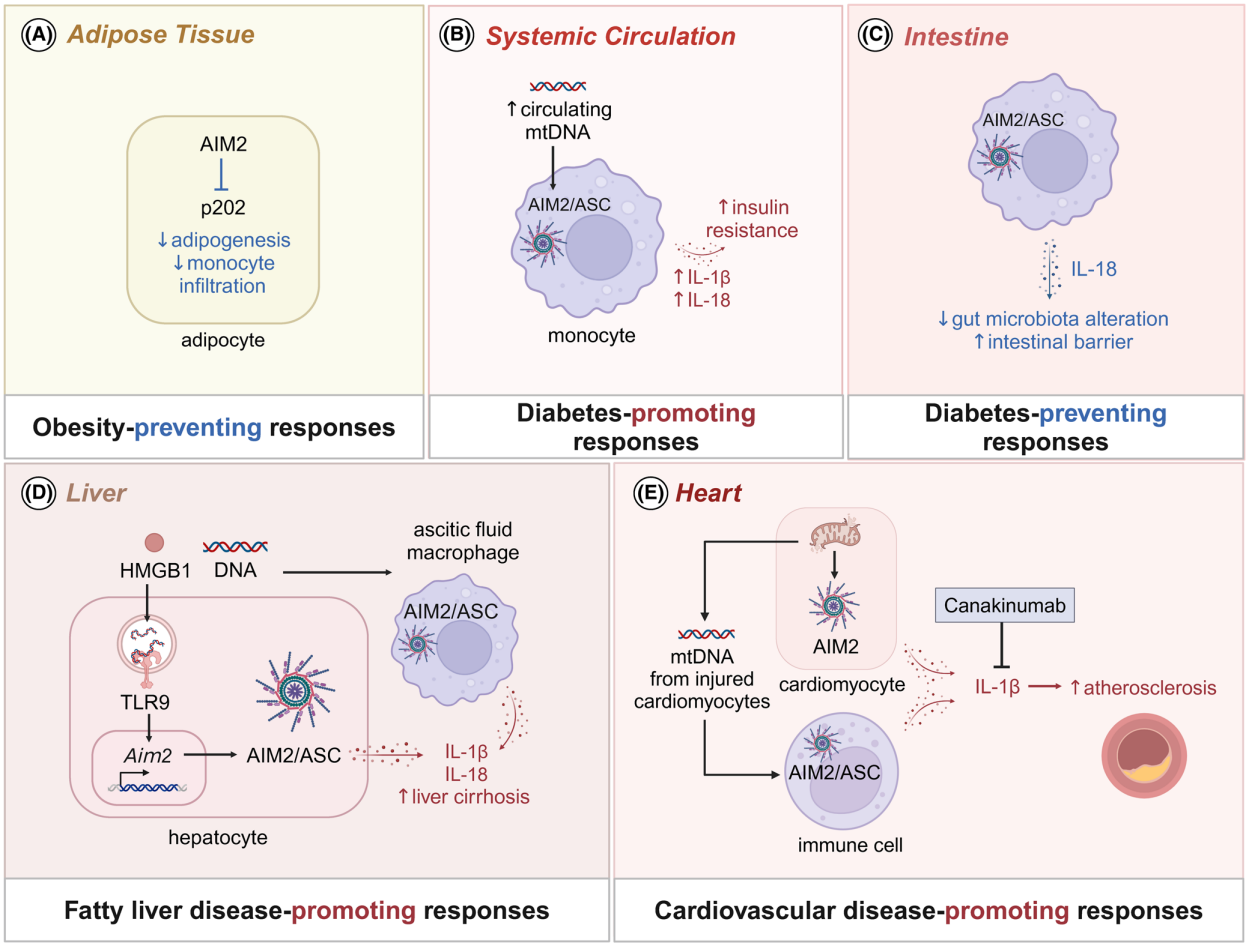
Role of AIM2 signaling in metabolic and cardiovascular diseases. (A) AIM2 inhibits the protein p202 to suppress adipogenesis and monocyte infiltration in adipose tissue. (B) Circulating mitochondrial DNA (mtDNA) activates the AIM2 inflammasome in monocytes, leading to the release of IL‐18 and IL‐1β. These cytokines promote insulin resistance and contribute to the development of type 2 diabetes (T2D). (C) In the intestines, AIM2‐mediated IL‐18 mitigates alteration in the gut microbiota, maintains the integrity of the gut barrier, and protects against the T1D development. (D) In liver, TLR9 activation by DNA and HMGB1 upregulates AIM2 expression and promotes AIM2 inflammasome activation in both immune and non‐immune cells. This activation leads to the release of pro‐inflammatory cytokines IL‐1β and IL‐18, aggravating liver damage and cirrhosis. (E) In the heart, mtDNA, oxidative DNA damage, and DNA replication stress activate the AIM2 inflammasome in cardiomyocytes and immune cells. The AIM2 inflammasome‐mediated secretion of IL‐1β in these cells drives atherosclerosis. The drug canakinumab inhibits IL‐1β signaling and reduces the incidence of recurrent cardiovascular events.

AIM2 plays a crucial role in energy metabolism and glucose homeostasis (Figure 4A–E). Studies have shown that mice deficient in AIM2 develop spontaneous obesity and exhibit impaired glucose homeostasis, enhanced adipogenesis, and inflammatory responses,^115^ indicating that AIM2 prevents metabolic syndrome. In stark contrast to AIM2, ablation of key components of the inflammasome, such as NLRP1, NLRP3, ASC, caspase‐1, and IL‐1β, protects mice from HFD‐induced obesity and insulin resistance.^116^, ^117^, ^118^ The levels of caspase‐1 activity in adipose tissue or circulating IL‐1β are similar in obese WT and *Aim2* ^−/−^ mice,^115^ suggesting that AIM2 prevents obesity independently of its inflammasome activity. Indeed, the increased adipogenesis in *Aim2* ^−/−^ mice is mediated through upregulation of protein 202 (p202), a homolog of human IFI16. Knocking down of *Ifi202b* in *Aim2* ^−/−^ cells rescues the adipogenesis^115^ (Figure 4A).

Inflammasome dependent cytokines IL‐1β and IL‐18 are known to drive insulin resistance and T2D.^119^ The expression of AIM2 in monocytes and levels of circulating mitochondrial DNA are higher in T2D patients compared to healthy controls. Increased circulating mitochondrial DNA is positively corelated with IL‐1β levels and the pathology of T2D.^120^ Stimulation of macrophages with mitochondrial DNA from T2D patients triggers the release of IL‐1β and IL‐18, which is dependent on the AIM2 inflammasome^18^ (Figure 4B). Despite heightened activity of AIM2 in T2D, AIM2‐mediated IL‐18 plays a protective role in the progression of streptozotocin‐induced T1D by mitigating alterations in gut microbiota and reinforcing intestinal barrier function^121^ (Figure 4C). Therefore, AIM2 exhibits a dual role in diabetes.

AIM2 has multifaceted roles in metabolic disorders such as obesity and diabetes. In obesity, AIM2 appears to suppress adipogenesis and preserve glucose homeostasis independently of its inflammasome activity. In T2D, AIM2 exacerbates insulin resistance through its inflammasome‐dependent cytokines, while in T1D, it provides a protective effect against disease progression by maintaining gut integrity. These insights highlight the complex and context‐dependent functions of AIM2, providing therapeutic insights for managing metabolic diseases.

### 5.2 AIM2 in fatty liver disease

AIM2 plays a significant role in the progression of fatty liver disease, including NAFLD and NASH. In mice, administration of HFD or methionine/choline deficient diet (MCD) augments hepatic expression of AIM2, caspase‐1, IL‐1β, and IL‐18,^122^, ^123^ indicating AIM2 inflammasome likely contributes to the progression of NAFLD and NASH. AIM2 and TLR9 signaling cooperate to exacerbate steatohepatitis. Endogenous DAMPs such as DNA and HMGB1 activate TLR9 signaling, upregulating AIM2 expression in a MyD88‐dependent manner in both immune and non‐immune cells of the liver^123^ (Figure 4D). Despite AIM2 upregulation in both cell types, mice deficient in MyD88 in bone‐marrow exhibit reduced liver injury, suggesting that AIM2 signaling in immune cells primarily aggravate liver damage.^123^ Ascitic fluid‐derived macrophages from cirrhotic patients show constitutive activation of the AIM2 inflammasome compared to PBMCs or macrophages from healthy controls.^124^ The secretion of IL‐1β and IL‐18 from these macrophages correlates with the severity of liver cirrhosis.^124^ AIM2‐mediated hepatocyte pyroptosis triggered by DNA release from lipid‐damaged mitochondria DNA also contributes to NAFLD.^22^ Stimulating AML‐12 liver cells with lipid and LPS triggers AIM2 inflammasome‐mediated pyroptosis. Silencing of IRF1, which is involved in AIM2 inflammasome activation^109^ and transcription of the mitochondrial DNA synthesis rate‐limiting enzyme CMPK2,^125^ blocks lipid and LPS‐induced AIM2 inflammasome activation.^22^ These findings imply that diet‐induced mitochondrial damage activates AIM2 inflammasome‐mediated pyroptosis and cytokine release in both macrophages and hepatocytes, contributing to the progression of liver disease (Figure 4D).

Certain host proteins in the liver antagonize AIM2 inflammasome activation. Overexpression of farnesoid X receptor (FXR), (NSAID)‐activated gene‐1 (NAG‐1), and growth differentiation factor‐15 (GDF15) ameliorates liver steatosis, reduces lipogenesis, and enhances lipolysis and fatty acid β‐oxidation, while concurrently suppressing AIM2 inflammasome activation.^23^, ^126^ Whether the hepatoprotection shown by these factors is truly mediated by negative regulation of the AIM2 inflammasome requires further investigation.

While the inflammasome‐dependent function of AIM2 in hepatocytes and macrophages in the liver may be harmful in conditions such as steatohepatitis, liver injury, and cirrhosis, AIM2 confers protection against drug‐induced hepatoxicity.^14^ During acute liver damage with acetaminophen overdose, AIM2 triggers autophagy by increasing the expression of LC3B and decreasing the expression of p62, Beclin1, and phosphorylated mTORC1.^14^

AIM2 plays a complex role in liver diseases, contributing to the progression of NAFLD and NASH through its inflammasome activity and cooperation with TLR9 signaling. However, AIM2 also shows protective roles in certain contexts, such as drug‐induced liver damage through autophagy.

### 5.3 AIM2 in cardiovascular disease

AIM2 signaling contributes to the development of various cardiovascular diseases, such as atherosclerosis, abdominal aortic aneurysm (AAA), and heart failure. In atherosclerosis, AIM2 and its ligand dsDNA are increased in macrophages at advanced disease stages in HFD fed ApoE‐deficient mice. The deletion of AIM2 rescues *Apoe* ^−/−^ mice from atherosclerosis.^127^ AIM2 blockade with ODN lowers IL‐1β and IL‐18 levels, reduces SMC death and necrotic core expansion, and increases collagen deposition and fibrous cap thickness, thus stabilizing atherosclerotic plaques and preventing its rupture.^127^ AIM2 inflammasome‐dependent cytokines IL‐1β^128^ and IL‐18^129^ contribute to atherosclerosis plaque progression and instability (Figure 4E). The CANTOS trial provides strong support for the significance of IL‐1β in atherosclerosis, as therapeutic IL‐1β neutralization in patients with established atherosclerotic disease significantly reduces the incidence of recurrent cardiovascular events.^130^ AAA patients exhibit increased AIM2 expression in peripheral granulocytes, monocytes, and B and T lymphocytes.^131^ Additionally, components of AIM2 inflammasome are increased in infiltrating lymphoid cells in the outer tunica media and adventitia of aortic samples from AAA patients,^132^ suggesting the involvement of AIM2 signaling in immune cells during aortic aneurysm.

Cardiac dysfunction associated with diabetes is known as diabetic cardiopathy (DCM).^133^ Mortality rates after myocardial infarction are significantly increased in T2D patients. T2D aggravates heart failure after myocardial infarction through defective mitophagy, exaggerated inflammasome activation, cell death, and IL‐18 secretion. In T2D mice, AIM2 expression is upregulated in both cardiomyocytes and macrophages.^133^ Silencing AIM2 improves cardiac survival and function in STZ‐induced diabetic rat models.^134^ During myocardial inflammation and dilated cardiomyopathy due to depletion of carnitine acetyltransferase (CRAT), type I IFN responses increase AIM2 expression and AIM2 inflammasome activation in cardiomyocytes.^135^ These pathologies are reversed by the deletion of AIM2 or caspase‐1, showing an inflammasome‐dependent role.

AIM2 signaling contributes to atherosclerotic cardiovascular disease associated with clonal hematopoiesis,^136^, ^137^, ^138^ which arises from somatic mutations that provide hematopoietic cells a proliferative advantage. The JAK2^V617F^ (JAK2^VF^) mutation, which increases JAK–STAT signaling, is a gain‐of‐function mutation that is associated with the increased risk for premature coronary heart disease.^139^ Atherosclerotic lesions in *Jak2* ^VF^ mice show increased expression of AIM2, and deletion of AIM2 or GSDMD reverses atherosclerosis.^136^ Moreover, deletion of caspase‐1 or GSDMD inhibits macrophage proliferation and necrotic cores in atherosclerotic lesions in mice expressing *Jak2* ^VF136^. Therefore, oxidative DNA damage and DNA replication stress caused by increased proliferation and glycolytic metabolism in *Jak2* ^VF^ macrophages leads to activation of the AIM2 inflammasome, aggravating atherosclerosis.

Overall, AIM2 signaling drives the pathogenesis of cardiovascular diseases such as atherosclerosis, AAA, and heart failure. Elevated AIM2 activity exacerbates these conditions by promoting inflammation, cell death, and plaque instability. Therapeutic targeting of AIM2 and its downstream pathways holds significant potential for stabilizing atherosclerotic plaques, reducing inflammation, and improving cardiac function, thereby offering promising strategies for treating these cardiovascular diseases.

## 6 INTERFERON GAMMA INDUCIBLE PROTEIN 16 (IFI16)

IFI16 is a crucial component of the innate immune response, belonging to the PYHIN (Pyrin and HIN domain‐containing) protein family. Encoded by the *IFI16* gene in humans, it serves as a cytosolic DNA sensor for the production of type I IFN responses. This ability to detect foreign or damaged DNA within the cell makes IFI16 a key player in the defense against viral infections and threats to genomic integrity. IFI16 is distinguished from other PYHIN family members by possessing two HIN (HIN‐A and HIN‐B) domains.^140^, ^141^ These domains are separated by a spacer region rich in serine, threonine, and proline residues. The length of this spacer region is regulated by alternative mRNA splicing, resulting in three isoforms of IFI16 (A, B, and C).^142^ The B isoform is the most abundantly expressed in human fibroblasts, epithelial cells, macrophages, and T cells. Initially identified as a nuclear protein, IFI16 is also found in the cytoplasm of many cell types, with its intracellular trafficking likely influenced by posttranslational modifications. IFI16 contains a nuclear localization signal in its N‐terminal region, facilitating its nuclear import.^143^

In mice, the PYHIN family includes over 10 members, with IFI204 and IFI202 closely resembling human IFI16 in molecular architecture and sequence homology in the PYD and HIN domains.^144^ These proteins, like IFI16, are induced by IFN‐α treatment and exist in phosphorylated forms in both the cytoplasm and nucleus. IFI16 can bind dsDNA in a sequence non‐specific manner, with a 16 bp DNA fragment sufficient for binding to four HIN‐B domains. Compared to AIM2, another PYHIN protein, IFI16 has significantly lower DNA‐binding affinity in its HIN‐B domain, which likely explains the requirement for two HIN domains in IFI16.^145^ Beyond dsDNA, IFI16 can bind various nucleotide structures, including superhelical and cruciform DNA, single DNA strands, and incomplete DNA reverse transcripts found in abortive HIV‐infected cells.^144^

### 6.1 IFI16 in obesity and diabetes

**FIGURE 5:**
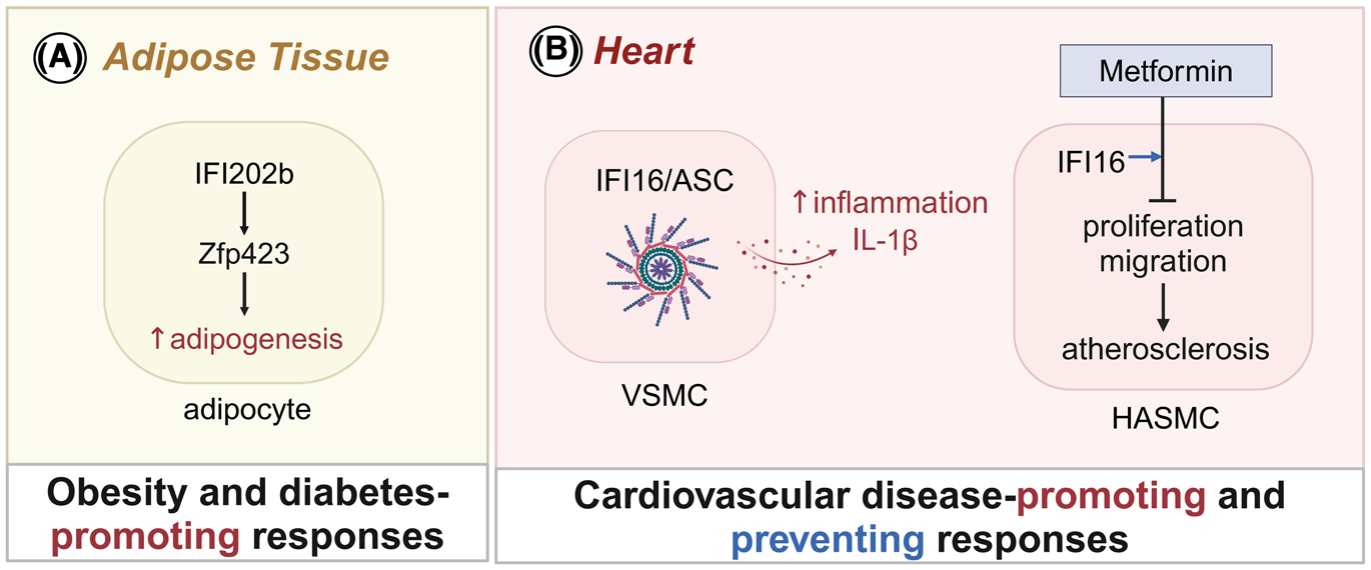
Role of IFI16 signaling in metabolic and cardiovascular diseases. (A) IFI202b, a homolog of human IFI16, promotes upregulation of the transcription factor zinc finger protein 423 (ZFP423), which is essential for adipogenesis. (B) In the heart, IFI16 interacts with ASC to assemble the IFI16 inflammasome in vascular smooth muscle cells (VSMCs), triggering cell death and the release of IL‐1β. The inflammatory responses driven by the IFI16 inflammasome promote cardiovascular diseases. IFI16 enhances Metformin's ability to suppress proliferation and migration of VSMCs, demonstrating its protective role in atherosclerosis.

IFI16 and its murine homologs, particularly IFI202b, have been implicated in the development and regulation of obesity. Expression of IFI16 is elevated in the adipose tissue of obese children. High levels of IFI16 in human adipose tissue are associated with larger adipocyte size and reduced insulin‐stimulated glucose uptake, suggesting a link between IFI16 expression and metabolic dysfunction in obesity.^146^ The mouse *Ifi205* and *Ifi202b* are closely related to human IFI16 and have been studied extensively to understand their roles in obesity. IFI202b has been identified as a putative obesity gene through positional cloning.^147^ It is significantly upregulated in New Zealand Obese (NZO) mice, which are commonly used as a model for studying obesity and metabolic syndrome.^115^ C57BL/6 mice expressing the genomic region containing the *Ifi200* gene cluster from NZO mice develops obesity and insulin resistance.^148^ Overexpression of IFI202b in mice leads to obesity and hepatic insulin resistance. Both IFI16 and IFI202b promotes adipocyte differentiation and lipid storage (Figure 5A). Suppressing IFI202b and IFI16 in pre‐adipocytes impairs adipocyte differentiation and reduces lipid accumulation. This effect is mediated by the downregulation of the transcription factor zinc finger protein 423, which is essential for adipogenesis.^146^ IFI205, which is minimally expressed in young adipocytes, is abundant in the perinuclear region and cytoplasm of adult adipocytes. Its expression is regulated by the transcription factor IRF7.^149^ Increased adipogenesis and inflammation in *Aim2* ^−/−^ mice are mediated through the upregulation of *Ifi202b*. Silencing *Ifi202b* in these mice blocks adipogenesis in stromal vascular fractions and reduces inflammation in macrophages.^115^ Type I IFN signaling involves in upregulation of *Ifi202b* in the absence of AIM2 in splenocytes.^150^ However, this mechanism of *Ifi202b* upregulation does not seem to operate in adipose tissue of *Aim2* ^−/−^ mice considering the undetectable levels of circulating or local type I IFNs, suggesting that alternative signaling pathways exist in metabolic tissues.^115^ It is important to note that *Ifi202b* is not transcribed in C57BL/6 mice due to a deletion of the first exon and the 5′‐regulatory regions. This genetic variation must be taken into account when interpreting findings from studies involving different mouse strains.^147^, ^148^

IFI16 and its murine homologs, particularly IFI202b, play significant roles in the development of obesity through their effects on adipocyte differentiation, insulin resistance, and inflammation. The upregulation of these proteins in adipose tissue is associated with adverse metabolic outcomes, making them attractive targets for further research in obesity and related metabolic disorders.

### 6.2 IFI16 in cardiovascular disease

IFI16 is involved in both promoting and mitigating cardiovascular pathologies, depending on the context and the specific signaling pathways activated. This dual role is primarily observed in conditions such as AAA and the cardioprotective effects of certain medications.

IFI16 has been implicated in the pathogenesis of AAA, a condition characterized by the dilation and potential rupture of the abdominal aorta. The expression of IFI16 is increased in aortic tissue from patients with AAA and in vascular smooth muscle cells (VSMCs) stimulated with angiotensin‐II.^151^ Silencing IFI16 and ASC inhibits angiotensin‐II‐induced cell death and IL‐1β release in VSMCs, indicating involvement of IFI16‐ASC inflammasome pathway in mediating inflammatory responses in AAA^151^ (Figure 5B). Therefore, selectively inhibiting the inflammasome‐dependent functions of IFI16 could reduce inflammation and vascular damage. Conversely, IFI16 promotes the cardioprotective effects of metformin, a widely used anti‐diabetic drug. Metformin's ability to inhibit cell proliferation and migration of human aortic SMCs is diminished when IFI16 is silenced,^152^, ^153^ indicating IFI16's protective role against vascular remodeling and atherosclerosis (Figure 5B). Therefore, enhancing IFI16 activity could improve the efficacy of cardioprotective drugs like metformin.

IFI16 plays contrasting roles in cardiovascular disease, acting both as a promoter of inflammation and cell death in AAA and as a mediator of cardioprotective effects in response to metformin. This dual functionality underscores the importance of context‐specific targeting of IFI16 for therapeutic interventions in cardiovascular diseases.

## 7 DNA‐DEPENDENT PROTEIN KINASE (DNA‐PK)

DNA‐PK is a trimeric complex essential for maintaining genomic stability and regulating critical cellular processes. Composed of a catalytic subunit (DNA‐PKcs) and DNA‐binding subunits Ku70 and Ku80, DNA‐PK primarily facilitates the nonhomologous end joining (NHEJ) pathway, a major mechanism for repairing double‐stranded DNA breaks (DSBs). In this process, Ku70 and Ku80 bind to DNA breaks, recruiting DNA‐PKcs to the site of damage to initiate repair. Disruption of any component of the DNA‐PK complex in mice results in premature aging and immunodeficiency, highlighting its fundamental role in preserving genetic stability and immune function.^154^ Severe combined immunodeficiency (SCID) mice, which harbor a mutation in DNA‐PK, have impaired lymphocyte development.^155^ Beyond its well‐known role in DNA repair, DNA‐PK influences transcription, DNA replication, immunity, and cellular metabolism. As a PRR for DNA viruses, DNA‐PK promotes chemokine secretion and interferon activation in response to viral DNA.^156^ It participates in STING‐dependent and independent DNA sensing pathways to trigger robust and broad antiviral responses.^157^ Ku70 specifically recognizes dsDNA and induces interferon activation. This dual role in both DNA repair and immune signaling underscores its versatility and importance in cellular defense mechanisms. DNA‐PK activation by CpG‐ODN in macrophages triggers the AKT signaling pathway, which is crucial for cell survival.^158^ These functions highlight DNA‐PK's integral role in the innate immune response and its potential as a target for therapeutic interventions.

### 7.1 DNA‐PK in obesity and diabetes

**FIGURE 6:**
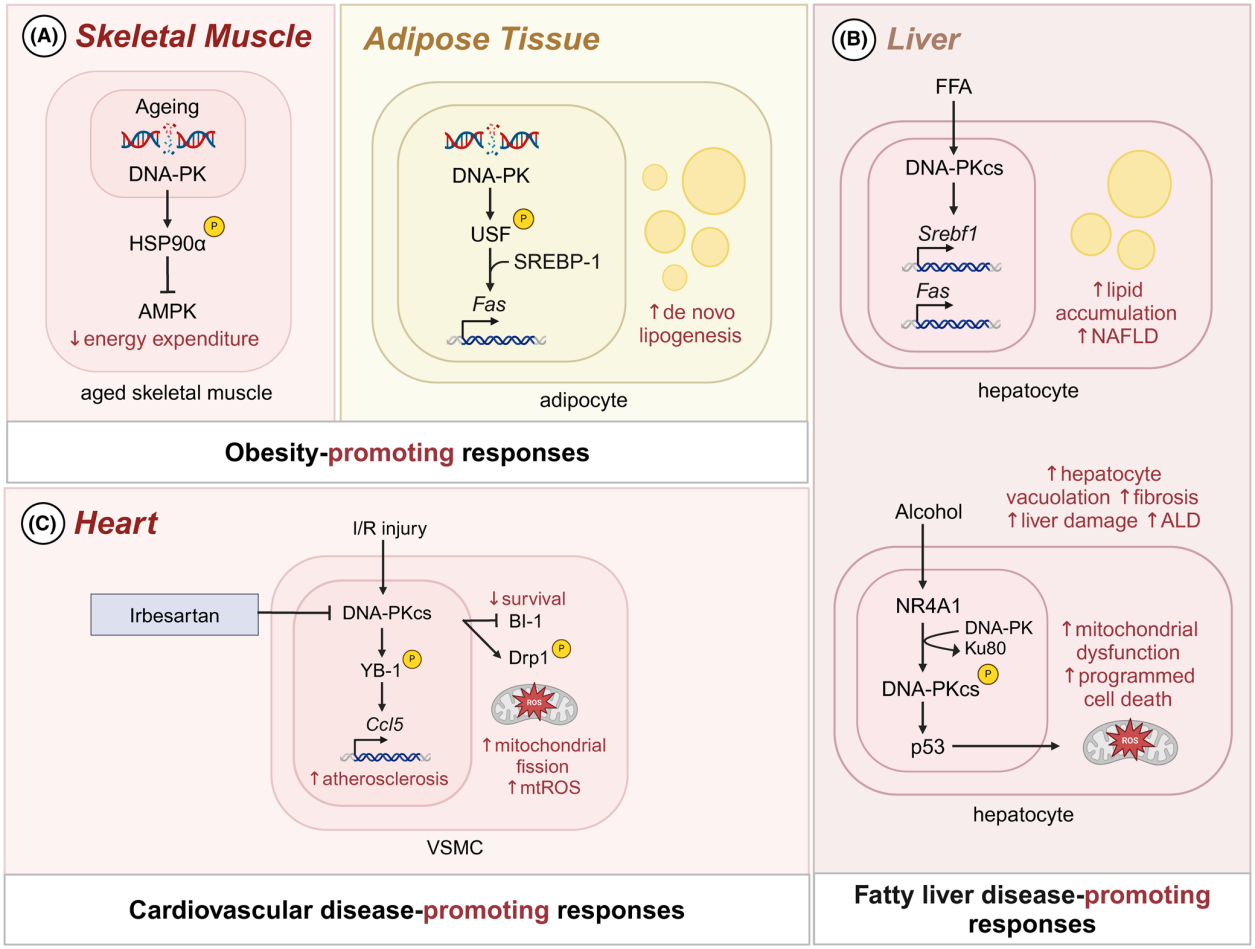
Role of IFI16 signaling in metabolic and cardiovascular diseases. (A) In skeletal muscle, DNA‐PK phosphorylates heat shock protein 90 (HSP90α), inhibiting AMPK activity and leading to decreased energy expenditure. In adipose tissue, DNA‐PK phosphorylates upstream stimulatory factor (USF), which recruits sterol regulatory element‐binding protein 1 (SREBP‐1) to the fatty acid synthase (FAS) promoter, enhancing lipogenesis. DNA‐PK mediated decreased energy expenditure and increased lipogenesis promote to the development of obesity. (B) In the liver, free fatty acids (FFAs) activate the catalytic subunit of DNA‐PK (DNA‐PKcs), which upregulates SREBP‐1 and FAS, leading to lipid accumulation and contributing to the development of non‐alcoholic fatty liver disease (NAFLD). Alcohol induces the phosphorylation of DNA‐PKcs via the orphan nuclear receptor (NR4A1). Phosphorylated DNA‐PKcs dissociates from Ku80, activates tumor suppressor gene p53, and initiates mitochondrial dysfunction and programmed cell death. The resulting hepatocyte damage leads to progression of alcoholic liver disease (ALD). (C) In the heart, ischemia–reperfusion (IR) injury stress induces DNA‐PKcs activation, which suppresses the pro‐survival molecule Bax inhibitor‐1 (BI‐1) by downregulating its expression and promoting its degradation. DNA‐PKcs phosphorylates the transcription factor Y‐box binding protein‐1 (YB‐1), which subsequently induces the expression of the chemokine CCL5. This process contributes to the recruitment of immune cells and initiates inflammatory responses. The anti‐hypertensive drug irbesartan reduces expression of DNA‐PKcs, thereby protecting against mitochondrial damage and reactive oxygen species (ROS) production.

DNA‐PK plays a complex role in development of obesity and diabetes, impacting various metabolic pathways. Loss of DNA‐PK activity has been shown to protect against diet‐induced obesity and insulin resistance, as well as age‐related loss of mitochondria and physical fitness in mice.^159^ SCID mice, which carry a leaky mutation in DNA‐PK, resist weight gain when fed a HFD. Furthermore, conditional deletion of DNA‐PK in skeletal muscle or administration of DNA‐PK inhibitors in WT mice leads to decreased weight gain and improved glucose tolerance and insulin sensitivity.^159^ These reduced obesity‐related phenotypes in SCID mice are not associated with lymphocyte deficiency since *Rag1* ^−/−^ mice, which also lack functional B and T lymphocytes, do not exhibit the same resistance to obesity on an HFD,^160^ indicating that the lymphocyte deficiency alone does not account for the metabolic phenotype observed in SCID mice. In contrast to the beneficial effects observed in SCID mice or with DNA‐PK inhibitors, DNA‐PKcs^−/−^ mice, which completely lack DNA‐PK activity, undergo accelerated aging.^161^ The paradox might be explained by the incomplete inhibition of DNA‐PK in SCID mice or with pharmacological inhibitors, leaving residual DNA‐PK activity that may provide some protection against naturally occurring DSBs. Aged macaques and mice show increased levels of DNA‐PK and HSP90α phosphorylation in skeletal muscle compared to younger animals. Skeletal muscle from older mice also exhibits higher levels of γ‐H2AX, a marker for DNA DSBs, suggesting that aging increases DNA damage in skeletal muscle. Older mice lacking DNA‐PK specifically in skeletal mice show lower phosphorylation of HSP90α but increased activation of AMPK and its substrate acetyl‐CoA carboxylase, indicating that DNA‐PK‐mediated phosphorylation of HSP90α suppresses AMPK activity.^159^ AMPK activation is protective against diet‐induced obesity and plays a central role in regulating metabolic diseases^162^ (Figure 6A).

Furthermore, the rise in mitochondrial reactive oxygen species (ROS) with aging leads to a decline in mitochondrial function in skeletal muscle, contributing to metabolic dysfunctions such as insulin resistance and T2D. Increased AMPK activity is beneficial as it decreases visceral fat, increases mitochondrial biogenesis, and enhances energy production in skeletal muscle, whereas loss of AMPK activity impairs glucose uptake. DNA‐PK is also involved in lipogenesis by promoting the transcription of fatty acid synthase (FAS), a central enzyme in de novo lipogenesis. Under fed conditions, DNA‐PK phosphorylates upstream stimulatory factor (USF), which recruits SREBP‐1 to the FAS promoter, enhancing lipogenesis^163^, ^164^ (Figure 6A).

The intricate role of DNA‐PK in metabolic regulation underscores its potential as a therapeutic target for obesity and diabetes, with partial inhibition offering benefits without the adverse effects associated with complete loss of activity. Moreover, the dual influence of DNA‐PK on inflammation and mitochondrial function highlights the need for a balanced approach in modulating its activity to mitigate age‐related metabolic dysfunctions.

### 7.2 DNA‐PK in fatty liver disease

DNA‐PK is intricately involved in the development and progression of both NAFLD and ALD. In NAFLD, DNA‐PK's role in promoting lipogenesis is particularly significant. DNA‐PKcs, the catalytic subunit, mediates free fatty acid‐induced lipid accumulation in hepatocytes, contributing to the pathogenesis of NASH.^165^ This process underscores DNA‐PK's involvement in the metabolic disturbances that characterize NAFLD, wherein excessive fat buildup in the liver leads to inflammation and, potentially, fibrosis (Figure 6B).

In ALD, DNA‐PKcs appears to have a cell death‐promoting effect that exacerbates liver damage. Under normal physiological conditions, DNA‐PKcs interacts with Ku80 to initiate DNA repair, maintaining genomic stability and cell survival.^166^ However, prolonged alcohol exposure leads to the phosphorylation of DNA‐PKcs by the orphan nuclear receptor NR4A1, resulting in its dissociation from Ku80.^167^, ^168^ This shift prompts DNA‐PKcs to activate p53, thereby initiating programmed cell death via mitochondrial pathways^169^ (Figure 6B). DNA‐PKcs‐induced p53 phosphorylation drives hepatic mitochondrial dysfunction by activating mitochondrial fission and repressing mitophagy (Figure 6B). In alcoholics, hepatocytes exhibit swollen mitochondria, mitochondrial DNA breaks, and ROS overproduction, all indicative of mitochondrial damage linked to ALD.^168^ Mice chronically fed ethanol show increased DNA‐PKcs phosphorylation in liver tissues, further associating DNA‐PKcs with ALD pathogenesis. Accordingly, mice lacking DNA‐PKcs in the liver demonstrate improved disease outcomes, evidenced by decreased hepatocyte vacuolation, reduced markers of liver damage, lower lipid droplet accumulation, and diminished fibrosis. The absence of NR4A1 leads to reduced phosphorylation of DNA‐PKcs and p53, which attenuates hepatocyte vacuolation, fibrosis, steatosis, and liver damage in response to alcohol treatment (Figure 6B). Moreover, DNA‐PKcs and NR4A1 are implicated in the broader context of fatty liver disease and hepatitis.^170^, ^171^ Increased levels of NR4A1 may pivot DNA‐PKcs from its DNA repair function to a role in cell death, suggesting a critical shift in liver disease progression pathways.

The involvement of DNA‐PK in these liver diseases highlights its dual roles in promoting lipid accumulation and triggering cell death, depending on the pathological context. Understanding the precise mechanisms through which DNA‐PKcs and its interacting partners, like NR4A1 and p53, influence liver pathology could lead to more effective treatments for these chronic liver conditions.

### 7.3 DNA‐PK in cardiovascular disease

DNA‐PK is increasingly recognized for its role in the pathogenesis of various cardiovascular diseases, including cardiomyopathy, heart failure, cardiac ischemia–reperfusion (IR) injury, and hypoxic pulmonary hypertension. Activation of DNA‐PK, particularly its catalytic subunit (DNA‐PKcs), in response to pathological stress is a common feature in these conditions. In cardiomyocytes, DNA‐PKcs activation under oxidative stress conditions such as IR injury leads to mitochondrial damage and myocardial cell death.^172^, ^173^, ^174^ Studies have shown that cardiac IR stress induces DNA‐PK activation, which promotes myocardial damage by impairing mitochondrial homeostasis and mitophagy. In mouse models, cardiomyocyte‐specific knockout of DNA‐PKcs results in reduced infarct size, decreased cardiomyocyte death, and lower myocardial inflammation upon IR injury, suggesting that DNA‐PKcs exacerbates cardiac damage.^174^

Moreover, the pro‐survival molecule Bax inhibitor‐1 (BI‐1) is downregulated in failing human hearts and in mice subjected to IR challenge. This downregulation is not observed in mice lacking DNA‐PKcs in cardiomyocytes, indicating DNA‐PKcs acts as an upstream negative regulator of BI‐1. Phosphorylated DNA‐PKcs binds to BI‐1 for its degradation, and genetic inhibition of BI‐1 abolishes the protective effects seen in DNA‐PKcs‐deficient mice during IR injury^174^ (Figure 6C). In VSMCs, DNA‐PKcs promotes pathological phenotypes such as vascular remodeling in response to angiotensin II infusion. DNA‐PKcs facilitates VSMC dedifferentiation and proliferation, contributing to conditions like hypoxic pulmonary hypertension and atherosclerosis.^175^, ^176^ Anti‐hypertensive drugs like irbesartan can suppress DNA‐PKcs expression, thereby protecting against vascular remodeling by reducing mitochondrial fragmentation and ROS production through interactions with Drp1^177^ (Figure 6C).

DNA‐PKcs also plays a significant role in the development of atherosclerosis. It is highly expressed in the neointima of human atherosclerotic tissues, where it phosphorylates Y‐box binding protein‐1 (YB‐1), a transcription factor crucial for various biological processes.^178^ YB‐1 mediates the expression of CCL5 in VSMCs, contributing to neointimal formation in atherosclerosis‐prone mice^179^ (Figure 6C). Inhibition of DNA‐PK in mice reduces neointimal lesions following vascular injury, indicating its potential as a therapeutic target.^176^ Additionally, DNA‐PKcs has been implicated in cardiotoxicity induced by the chemotherapeutic drug doxorubicin. Doxorubicin treatment leads to the upregulation of TBC1D15, which interacts with DNA‐PKcs, causing its cytosolic retention and contributing to the development of dilated cardiomyopathy.^180^

In conclusion, DNA‐PKcs is a critical mediator in the pathogenesis of various cardiovascular diseases, from myocardial damage in IR injury to vascular remodeling and atherosclerosis. Its roles in promoting cell death and facilitating inflammation and VSMC proliferation underscore its importance as a potential therapeutic target. Identifying the specific mechanisms through which DNA‐PKcs interacts with proteins like BI‐1, Drp1, and YB‐1 could pave the way for developing targeted treatments for cardiovascular conditions, reducing the burden of these chronic diseases.

## 8 DEAD‐BOX HELICASE 41 (DDX41)

DDX41 is a member of the DEAD‐box protein family, characterized by the conserved motif Asp‐Glu‐Ala‐Asp (DEAD). DEAD‐box protein family proteins are ATP‐dependent RNA helicases that play crucial roles in various aspects of RNA metabolism, including transcription, splicing, ribosome biogenesis, and translation. DDX41 comprises several conserved domains typical of DEAD‐box proteins, such as the DEAD motif, a helicase C‐terminal domain, and ATP‐binding and hydrolysis sites.

One of the pivotal roles of DDX41 is its involvement in the immune responses during infection with DNA viruses and bacteria.^5^, ^6^ DDX41 acts as a cytosolic DNA sensor, leading to the activation of downstream signaling pathways and production of type I IFNs. DDX41 recognizes intracellular DNA as well as bacterial cyclic di‐GMP and cyclic di‐AMP to activate cGAS‐STING signaling. DDX41 utilizes its DEAD domain to recognize the two different ligands dsDNA and cyclic dinucleotides.^181^ While DDX41 is not directly targeted by IFN, the IFN‐inducible E3 ubiquitin ligase TRIM21 regulates DDX41 through Lys48‐linked ubiquitination and subsequent degradation.^182^ Additionally, BTK facilitates DDX41 activation through phosphorylation, enhancing its binding to STING.^183^

In addition to its roles in innate immunity, mutations in the DEAD domain of human DDX41 are associated with myelodysplastic syndrome (MDS) and acute myeloid leukemia (AML). DDX41 is necessary to control replication stress. Loss of DDX41, or pathogenic variants of DDX41 results in impaired unwinding of RNA–DNA hybrids which leads to increased R‐loop formation and DSB.^184^ MDS/AML DDX41 variants excessively activate cGAS‐STING signaling, which may be due to their reduced DNA unwinding activity impacting dsDNA ligand availability for cGAS binding.^185^

Although current research does not extensively cover the direct involvement of DDX41 in metabolic diseases such as obesity, diabetes, fatty liver diseases, or cardiovascular diseases, there are emerging lines of evidence suggesting its broader relevance. High‐risk heart failure patients have been found to have DDX41 variants.^186^ Loss of function of the transcription factor MEF2A, associated with increased risk for inherited coronary artery disease outcomes,^187^ could be exacerbated by aberrant IFN induction.^30^ Depletion of MEF2A leads to R‐loop accumulation and DDX41‐cGAS‐STING‐dependent inflammatory responses, highlighting a potential link between DDX41 and cardiovascular diseases.^188^ Overall, many lines of evidence highlight DDX41 as an upstream sensor of the cGAS‐STING pathway, implicating its relevance in cGAS‐STING‐dependent regulation of metabolic and cardiovascular diseases.

DDX41 is a multifaceted protein with essential roles in RNA metabolism and the innate immune response. Its significance in disease, particularly in hematologic malignancies, underscores the importance of continued research to unravel its complex functions and therapeutic potential. Moreover, emerging evidence suggests that DDX41 could be relevant in the regulation of metabolic and cardiovascular diseases, warranting further investigation into its broader clinical implications.

## 9 CONCLUSIONS, FUTURE PERSPECTIVES, AND OPEN QUESTIONS

Abnormal DNA from endogenous and exogenous sources marks metabolic disorders. Our discussion has highlighted the crucial roles of DNA sensors–TLR9, cGAS‐STING, AIM2, IFI16, DNA‐PK, and DDX41 in conditions like obesity, diabetes, fatty liver disease, and cardiovascular disease. These DNA sensors play complex roles, with studies showing both protective and detrimental roles depending on the cellular context and disease state.

The DNA sensors signal through downstream TBK1‐IRF3 or TBK1‐NF‐κΒ pathways, leading to the production of type I IFNs or pro‐inflammatory cytokines. Type I IFNs drive systemic insulin resistance in human subjects.^189^ Pegylated IFN‐α therapy in patients with hepatitis C viral infection is associated with insulin resistance. Genetic deficiency of IRF7, the critical transcription factor for induction of type I IFNs in pDCs, protects mice from HFD‐induced obesity.^190^ Clinically, an associated risk of developing metabolic disorders has already been established in SLE and psoriasis, where pDC‐derived type I IFNs play a central role in pathogenesis.^191^

There are several limitations in studies investigating the role of DNA sensors in metabolic and cardiovascular diseases. Key questions that need to be addressed include: (1) What are the specific contributions of different DNA sensors in various tissues and cell types in the pathogenesis of metabolic and cardiovascular diseases? Many studies lack cell type specificity, often relying on global deficiency of DNA sensors. This approach can obscure the distinct roles of DNA sensors in different cell types and tissues. The observed phenotype in whole‐body knockouts of a particular sensor reflects the cumulative impacts of its deletion across all tissue types. These cumulative effects may obscure individual tissue‐specific contributions, complicating the precise understanding of its role in metabolic disorders. Going further, it is imperative to develop more targeted methods for elucidating the cell‐specific contributions of DNA sensors. (2) What are the precise sources and triggers of abnormal DNA that activate these sensors? Furthermore, how do factors like oxidative stress, DNA replication stress, and DNase deficiencies contribute to this process? (3) If multiple DNA sensors can be activated simultaneously, does cross‐talk between these sensors exist? Understanding the interactions and regulatory mechanisms between different DNA sensors is crucial for a comprehensive understanding of their roles in disease. (4) How does a single cytokine, secreted downstream of DNA sensor activation, exert contrasting effects? Moreover, is it possible to selectively inhibit the detrimental actions of these cytokines while preserving their beneficial ones? (5) Can we identify a therapeutic window for modulating DNA sensor activity that maximizes therapeutic benefits and minimizes adverse effects in the treatment of metabolic and cardiovascular diseases?

By addressing these open questions, we can develop more precise and effective therapeutic strategies for metabolic and cardiovascular diseases, ultimately improving patient outcomes.

## CONFLICT OF INTEREST STATEMENT

There are no conflicts of interest to declare.

## Data Availability

Data sharing is not applicable to this article as no new data were created or analyzed in this study.
